# Copper nanoparticles/polyaniline/molybdenum disulfide composite as a nonenzymatic electrochemical glucose sensor

**DOI:** 10.1016/j.heliyon.2023.e21272

**Published:** 2023-10-26

**Authors:** Krishna Prasad Sharma, Miyeon Shin, Kyong Kim, Kyungmin Woo, Ganesh Prasad Awasthi, Changho Yu

**Affiliations:** a Department of Energy Storage/Conversion Engineering (BK21 FOUR), Jeonbuk National University, Jeonju, Jeollabuk-do, 54896, Republic of Korea; b Department of Rehabilitation Engineering, Daegu Hanny University, Gyeongsan, Gyeongsangbuk-do, 38609, Republic of Korea; c Division of Convergence Technology Engineering, Jeonbuk National University, Jeonju, Jeollabuk-do, 54896, Republic of Korea

**Keywords:** Glucose sensor, Sensitivity, Detection limit, Selectivity, Polymerization

## Abstract

A Cu@Pani/MoS_2_ nanocomposite was successfully synthesized via combined in-situ oxidative polymerization and hydrothermal reaction and applied to an electrochemical nonenzymatic glucose sensor. The morphology of the prepared Cu@Pani/MoS_2_ nanocomposite was characterized using FE-SEM and Cs-STEM, and electrochemical analysis was performed using cyclic voltammetry (CV), electrochemical impedance spectroscopy (EIS), and chronoamperometry techniques. Electrostatic interaction between Cu@Pani and MoS_2_ greatly enhanced the charge dispersion, electrical conductivity, and stability, resulting in excellent electrochemical performance. The Cu@Pani/MoS_2_ was used as an electrocatalyst to detect glucose in an alkaline medium. The proposed glucose sensor exhibited a sensitivity, detection limit, and wide linear range of 69.82 μAmM^−1^cm^−2^, 1.78 μM, and 0.1–11 mM, respectively. The stability and selectivity of the Cu@Pani/MoS_2_ composite for glucose compared to that of the potential interfering species, as well as its ability to determine the glucose concentration in diluted human serum samples at a high recovery percentage, demonstrated its viability as a nonenzymatic glucose sensor.

## Highlights

• Cu@Pani/MoS_2_ nanocomposite was prepared via in-situ oxidative polymerization followed by hydrothermal method.
• Cu@Pani/MoS_2_ nanocomposite exhibits flower like nanosheet morphology.
• The π-π interaction between MoS_2_ and Cu@Pani greatly enhance the sensitivity and selectivity of glucose sensor.
• The Cu@Pani/MoS_2_ composite reveal high sensitivity of 69.82 μAmM^−1^cm^−2^ and detection limit of 1.78 μM.

## Graphical abstract

**Figure:**
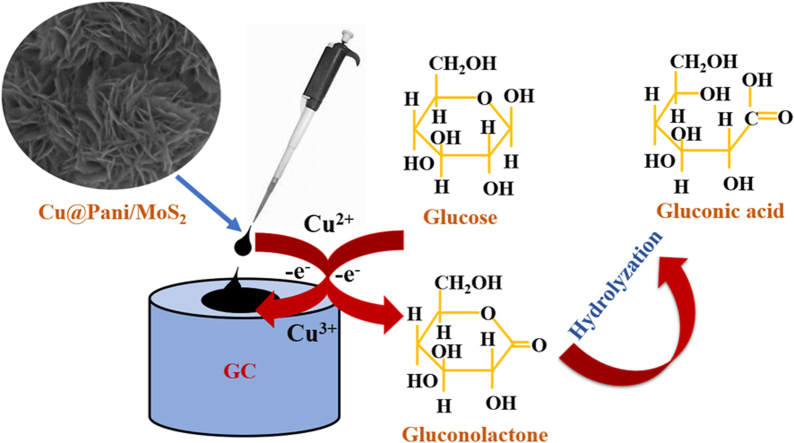

## 1 Introduction

Inadequate insulin secretion by pancreatic beta cells is a characteristic of diabetes mellitus, which is a metabolic disorder that can cause serious health issues and even death [[1], [2], [3]]. “According to the World Health Organization diabetes is a leading cause of blindness, kidney failure, heart attacks, strokes, and lower limb amputation” [4]. These associated health risks have led to an emerging interest in the advancement of straightforward early diagnosis and treatment techniques for diabetes mellitus [5]. To this end, researchers have gained considerable interest in electrochemical glucose sensors, because of their cost effectiveness, high sensitivity, high stability, and ease of handling during experiments [[6], [7], [8], [9]], Due to these advantages, highly-sensitive glucose sensors are used in diverse fields, such as medical diagnostics [10], agro-food technology [11], and pharmaceuticals [12]. Enzyme-based electrochemical sensors immobilize enzyme glucose oxidase (GO) on an electrode surface and exhibit high selectivity; however, they have shortcomings such as poor stability in pH, and temperature variation, high production cost, short lifetime of the enzyme, and low electrochemical signals attributed to the low energy transfer. In addition, the adsorption of protein molecules form the blood on the enzyme surface further reduces the utility of enzymatic sensors [13,14]. To overcome these problems, electrochemical non-enzymatic glucose sensors with characteristic features such as effective selectivity, high sensitivity, low detection limit, and better stability have been developed [[15], [16], [17]]. Nanomaterials composed of metals such as copper (Cu), iron (Fe), gold (Au), silver (Ag), manganese (Mn), nickel (Ni) and zinc (Zn) have been examined for their applicability in non-enzymatic glucose sensors. Among these**,** metal oxides and metal sulfides have been used owing to their large surface areas and high surface energies, which substantially increase electrocatalytic activity during the electrooxidation of glucose [14], [[18], [19], [20], [21], [22], [23]]. Copper nanoparticles (CuNPs) have gained considerable interest in electrochemical research because of their low cost, better electrocatalytic activity, biocompatibility, and stability [14,24]. To this end, numerous studies have focused on developing electrochemical nonenzymatic glucose sensors for fabricating nanomaterials with high porosity, large surface area, low detection limit, and excellent sensitivity [25,26]. Inorganic/organic support-catalyst based composite nanoparticles may be a suitable electrocatalyst material, because of their improved ion dispersion, enhanced electron transfer, and better stability between the electrode surface and electrolyte [[27], [28], [29], [30], [31]]. Conducting polymers have been used as an electrocatalyst support and metal nanoparticles stabilizing agent because of their intriguing redox properties, high stability, and ease of synthesis [[32], [33], [34]].

Polyaniline (Pani) conducting polymers have attracted research interest because of their redox properties. The surface area of a conducting polymer can be increased via synthesis in the presence of metal nanoparticles [[35], [36], [37]]. The synergistic properties of metal nanoparticles dispersed onto a polymer composite exhibits enhanced electrochemical properties [27,38]. Owing to its large surface area, and distinct physicochemical characteristics, molybdenum disulfide (MoS_2_), has been used in nanoelectronics, lithium-ion batteries [39], catalysis [40], supercapacitors [41,42], and sensors [43,44]. However, MoS_2_ also has disadvantages that inhibit its wider application, e.g., restacking, intrinsically low conductivity, and tendency to agglomerate. These issues can be resolved by doping metal nanoparticles with the conducting polymer and carbon based materials [1,45,46]. Novel nanocomposites based on conducting polymers, metal nanoparticles, and MoS_2_ featuring a unique structures and properties could significantly improve electrochemical nonenzymatic glucose sensors.

In previously studies, Yu et al. demonstrated that a NiCo_2_O_4_@PANI core-shell nanocomposite is relevant for sensitive glucose determination [47]; Yang et al. reported CuNPs deposited multi-walled carbon nanotube arrays for amperometric non-enzymatic glucose sensor [48]; and Belgherbi et al. used copper-polyaniline composite films for enzyme-free glucose sensors [6]. Further, Zheng et al. reported an electrochemical glucose sensor based on a copper nanoparticles/polyaniline/graphene composite [14]. Esmaeeli et al. reported a copper-oxide decorated polyaniline nanofiber applicable to non-enzymatic glucose sensors [49]. Moreover, Pratap et al. applied copper nanoparticles-supported mesoporous polyaniline (Cu/Meso-PANI) to nonenzymatic glucose sensor [50]. Chen et al. synthesized Cu-doped polyaniline through the H_2_O_2_-promoted oxidative polymerization of aniline [51]. To the best of the authors’ knowledge, this is the first study that presents a synthesis procedure combining in-situ oxidative polymerization, and a hydrothermal process for synthesizing a Cu@Pani/MoS_2_ nanocomposite. CuNPs were doped onto polyaniline during in-situ polymerization, nucleation, and growth during the hydrothermal reaction. The as-prepared Cu@Pani/MoS_2_ composite was loaded on a glassy carbon electrode, and it exhibited the excellent electrocatalytic performance, indicating its effectiveness for nonenzymatic glucose sensors.

## 2 Materials and methods

### 2.1 Chemicals and synthesis

Hydrochloric acid (Samchun), aniline (Alfa Aesar), copper sulfate anhydrous (Daejung), hydrogen peroxide (Samchun), trisodium citrate dihydrate (Shova chemicals), sodium molybdate dihydrate (Samchun), thiourea (Junsei) and human serum (Sigma- Aldrich H4522) were purchased and used directly without additional purification. Ultrapure double distilled water (Millipore, USA) was used for solution preparation throughout the experimental process.

### 2.2 Synthesis of Cu@Pani and Cu@Pani/MoS_2_

Cu@Pani was prepared using a slight modification to the method reported by Chen et al. [51], through the in-situ oxidative polymerization of aniline along with a copper salt. 50 mM aniline, 10 mM copper sulfate anhydrous, 34 mM trisodium citrate dihydrate (NaCt), and 50 mM hydrogen peroxide (H_2_O_2_) were added, to a 70 mL 1 M HCl solution, and the mixture was stirred for 24 h at 400 rpm to synthesize Cu@Pani. Similarly, for Cu@Pani/MoS_2_, 30 mM sodium molybdate and 100 mM thiourea were also added by following the above-mentioned procedure for Cu@Pani. After stirring 30 min, the solution was kept in a Teflon-lined hydrothermal vessel at 180 °C for 24 h. The obtained Cu@Pani and CuPani/MoS_2_ composites were separated using a centrifuge, washed in distilled water, and ethanol, and then dried at 70 °C for 12 h.

### 2.3 Electrode preparation

The following procedures were followed to prepare Cu@Pani and CuPani/MoS_2_ modified electrodes: Before loading the electrode material, a glassy carbon (GC) electrode, which has a diameter of 5 mm (A = 0.1963 cm^2^), was polished with 0.05, 0.3 and 1.0 μm alumina slurry, washed with water and ethanol, and allowed to dry at room temperature. 3 mg of both Cu@Pani and CuPani/MoS_2_ were dispersed separately in 200 μL isopropanol and 5 μL 5 % Nafion solution to prepare the ink solution. The GC electrode was modified by drop-casting 20 μL of the dispersed solution onto the electrode surface and allowed it to dry at room temperature.

### 2.4 Characterization

Field emission scanning electron microscopy (Ultrahigh FE**‐**SEM: SU8220 HITACHI, Japan) coupled with energy dispersive X**‐**ray spectroscopy (EDX) and Cs**‐**corrected scanning transmission electron microscopy (Cs**‐**STEM; JEOL/JEM**‐**ARM200F) were used to characterize the surface morphologies of the Cu@Pani and CuPani/MoS_2_ nanocomposites. The Fourier**‐**transform infrared spectroscopy (FTIR, PerkinElmer, Spectrum GX, USA) KBr plate method was used to record the FT-IR spectra in the transmission mode. The XRD patterns of Cu@Pani and Cu@Pani/MoS_2_ composites were examined by using an X**‐**ray powder diffractometer (XRD, Rigaku Co., Japan) with monochromatic Cu Kα radiation (λ = 1.54056 Å) at a Bragg angle (2θ) range of 5**‐**70°. X**‐**ray photoelectron spectroscopy (XPS) was performed using a Nexsa XPS Surface Analysis System (Thermo Fisher Scientific, UK) outfitted with a monochromatic Al Kα (1486.6 eV) X**‐**ray light source.

### 2.5 Electrochemical measurements

All electrochemical measurements including cyclic voltammetry (CV), electrochemical impedance spectroscopy (EIS) and chronoamperometry (i-t) were performed using a ZIVE SP2 potentiostat (Won ATech-ZIVE LAB). A GC electrode, Pt wire, and calomel electrode Hg_2_Cl_2_ were used as the working, counter, and reference electrodes, respectively. CV was performed with 0.1 M NaOH solution as the working electrolyte at various scan rates (5-100 mVs^−1^) and potential ranges between 0 and 0.8 V. Between 0.1 Hz and 100 KHz, EIS was conducted using a 1:1 mixture of 5 mM Fe (CN)_6_^3−/4-^ and 0.1 M KCl solution as the supporting electrolyte The Chronoamperometry was performed at + 0.5 V while stirring at 500 rpm.

## 3 Results and discussion

**Fig. 1:**
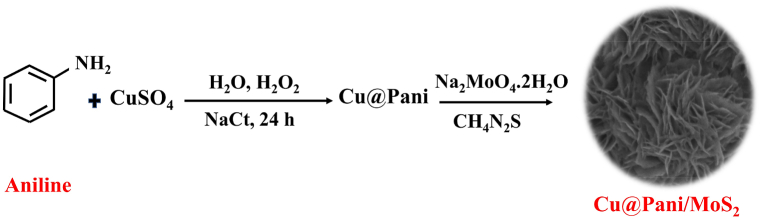
Scheme for the synthesis of Cu@Pani and Cu@Pani/MoS_2_ nanocomposites.

The processes used for Cu@Pani and Cu@Pani/MoS_2_ synthesis is illustrated in Fig. 1. The Cu@Pani/MoS_2_ composite was prepared through in-situ polymerization and hydrothermal reaction using hydrogen peroxide (H_2_O_2_) as a green oxidant followed by a hydrothermal reaction. During, the in-situ polymerization, H_2_O_2_ acts as the green oxidant that generates a green waste product, i.e., water. Further, CuNPs are doped into polyaniline, resulting Cu@Pani composite during polymerization. The oxidation potential of Cu (+0.34 V) is not sufficiently high to oxidize aniline [51], and therefore, we added H_2_O_2_ to the copper and aniline solution to promote the oxidation of aniline to polyaniline, which undergoes in-situ polymerization to form black precipitates of the copper polyaniline composite (i.e., Cu@Pani). Sodium molybdate and thiourea were reduced and sulfurized during a hydrothermal reaction to prepare Cu@Pani/MoS_2_ [51,52]. Simultaneously, after the addition of Nact and H_2_O_2_, the copper salt is reduced to produce CuNPs. Meanwhile, H_2_O_2_ acts as a oxidizing agent that initiates polymerization [53], and NaCt act as a reducing and stabilizing agent such that the nucleation and growth processes and polymerization occur in parallel [38,[54], [55], [56]]. The CuNPs were doped onto polyaniline during in-situ polymerization, and MoS_2_ nanosheets were grown on the surface of Cu@Pani during the hydrothermal reaction. The covalent linkage between polyaniline (N-atoms) and MoS_2_ occurs via metal (dπ) - nitrogen pπ bonding and bonding between nitrogen and sulfur atom. The linkage of MoS_2_ nanosheet and conjugated polyaniline occurs through π-π interaction between MoS_2_ nanosheet and conjugated polyaniline [57,58], as displayed in Fig. 1.

**Fig. 2:**
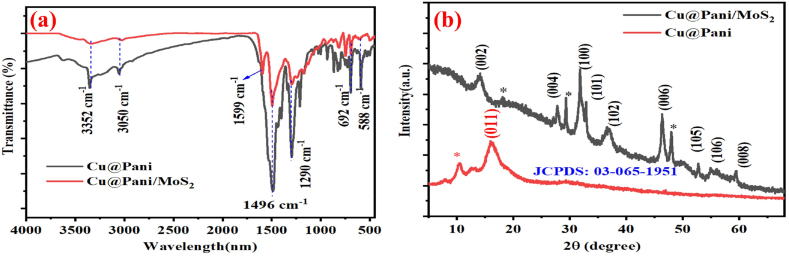
(a) FT-IR spectra (b) XRD spectra of Cu@Pani and Cu@Pani/MoS_2_, respectively.

The Cu@Pani and Cu@Pani/MoS_2_ FTIR spectra are shown in Fig. 2(a). The peaks located at 3352 and 3050 cm ^−1^ are attributed to the N–H stretching vibration. The FT-IR absorption peaks at 1599, 1496, and 1290 cm ^−1^ are attributed to the N = Q = N stretching of the quinine ring, C=C bonding in the conjugated polyaniline, and C–N bond vibration of aromatic benzene ring, respectively [51,59]. [60]. Similarly, the peak at 692 cm ^−1^ corresponds to the copper metal polyaniline linkage through the amino group of aniline [61]. The increased transmittance of Cu@Pani/MoS_2_ composite as compared to Cu@Pani confirmed the proper conjugation, and the weak band at 588 cm ^−1^ attributed to Mo–S bond vibration in Cu@Pani/MoS_2_ [37,62]. The Cu@Pani and Cu@Pani/MoS_2_ XRD spectra are shown in Fig. 2(b). The XRD patterns for Cu@Pani/MoS_2_ at 14.1^°^, 27.75^°^, 31.73^°^, 32.83^°^, 36.95^°^, 46.32^°^, 52.67^°^, 54.98^°^, and 59.42^°^ corresponds to (002), (004), (100), (101), (102), (006), (105), (106), and (008) crystal planes, respectively (JCPDS: 03-065-1951) [63]. Similarly, Cu@Pani diffraction at 16.08^°^ can be attributed to the (011) crystal plane, which is parallel to the long chain polymer of aniline [59,63,64]. The deviation of the peak position from the usual peak around the 2θ angle 10.37^°^ and the emergence of new peaks at 2θ angles 18.22^°^, 29.34^°^, and 48.07^°^, can be attributed to the scattered orientation of the parallel aniline chain as well as copper, trisodium citrate dihydrate and MoS_2_, respectively, as indicated by asterisk (*). The characteristic peaks of Cu@Pani disappeared in the final Cu@Pani/MoS_2_ composite, indicating the successful in-situ polymerization of aniline [65].

**Fig. 3:**
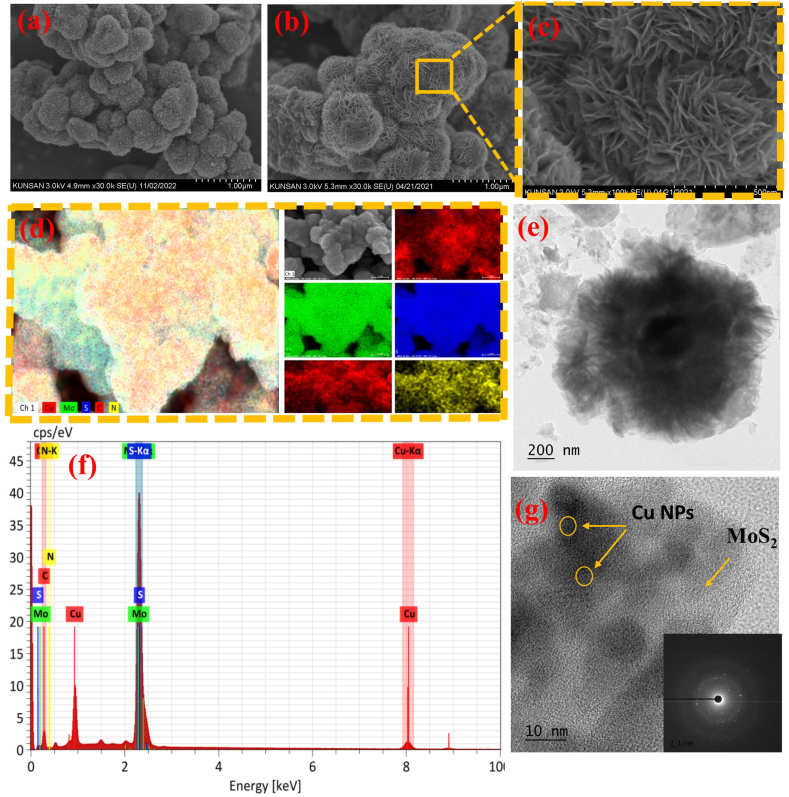
(a) FE-SEM of Cu@Pani and (b) Cu@Pani/MoS_2_, (c) enlarged FE-SEM image of Cu@Pani/MoS_2_, (d) EDX color mapping of Cu@Pani/MoS_2_, (e) Cs-STEM of Cu@Pani/MoS_2,_ (f) EDX spectrum of Cu@Pani/MoS, and (g) a high-resolution TEM of Cu@Pani/MoS_2_ with SEAD pattern.

The Cu@Pani and Cu@Pani/MoS_2_ composite FESEM and CS-TEM images were recorded to study the surface morphology, and they are presented in Fig. 3. As shown in the FE-SEM image of Cu@Pani, nanoflowers composed of Cu nanoparticles doped polyaniline showed a ball-like structure. The surface morphology of Cu@Pani/MoS_2_ revealed that MoS_2_ nanosheet were grew on the surface of Cu@Pani, as represented in Fig. 3 (a, b) [51,66]. The enlarged image of Cu@Pani/MoS_2_ in Fig. 3(c) shows a flower-like MoS_2_ nanosheet structure, which confirmed the formation of MoS_2_ nanosheets [67,68]. Cs-STEM imagery revealed a nanosheet structure with agglomerated MoS_2_, as shown in Fig. 3(e). Elemental color mapping revealed that Mo, Cu, N, S and C were homogeneously distributed over the composite 3(d). The EDX spectrum in Fig. 3(f) showed that the composite was composed of 28.49 % carbon, 24.22 % molybdenum, 25.08 % sulfur, 16.41 % copper, and 4.92 % nitrogen [69,70]. This information is presented in Fig. S3(c).

**Fig. 4:**
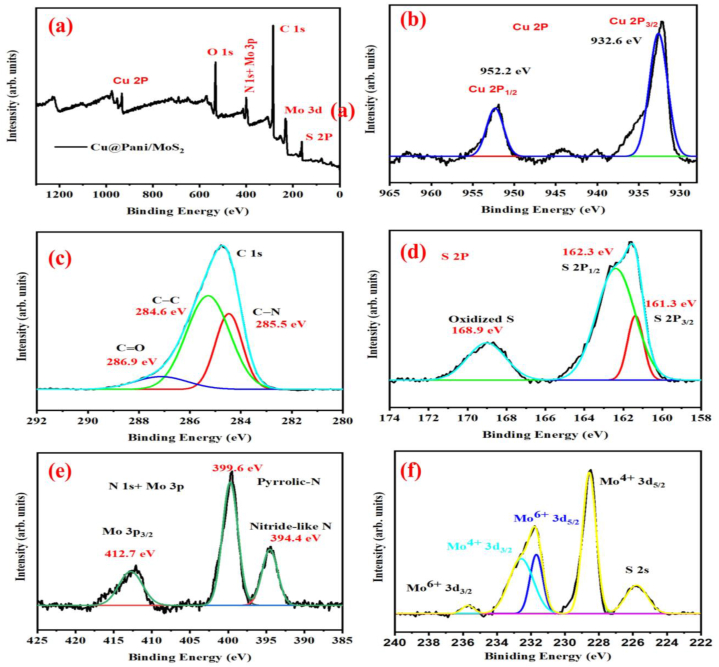
(a) XPS spectrum of Cu@Pani/MoS_2_, (b–f) Cu 2P, C 1s, S 2P, N 1s + Mo 3P, and Mo 3d in Cu@Pani/MoS_2_.

The chemical composition of the nanocomposite material was studied by using XPS, and the results are shown in Fig. 4(a). The broad scan XPS spectrum revealed the presence of copper (Cu), molybdenum (Mo), nitrogen(N), sulfur (S) and carbon (C) and oxygen (O) elements [3]. After deconvolution, peaks at 932.2 and 952.2 eV binding energies corresponds to Cu 2P_3/2_ and Cu 2P_1/2_ respectively, and the small peaks at 944 eV and 940 eV represent satellite peaks of copper, which indicate the paramagnetic behavior, of the Cu^2+^ oxidation state. The binding energy difference of 19.6 eV between the 2P_3/2_ and 2P_1/2_ states of Cu resembles the reported 20 eV binding energy difference, as shown in Fig. 4(b) [51]. [71,72]. The carbon peaks displayed in Fig. 4(c) fit at 284.6, 285.5 and 286.9 eV binding energies assigned for the C–C, C–N and C = O species [3,[73], [74], [75]]. Binding energies at 161.3, 162.3 and 168.9 eV shown in Fig. 4(d) are attributed to S 2P_1/2_, S 2P_3/2_ and oxidized sulfur, respectively [43,76,77]. The XPS spectrum shown in Fig. 4(e) with binding energies at 394.4, 399.6 and 412.7 eV are attributed to the nitride like –N, pyrrolic-N, and Mo 3P_3/2_ respectively [[78], [79], [80]]. Binding energy peaks at 225.8, 228.5, 331.6, 232.6 and 235.7 eV are responsible for S 2s, Mo^4+^ 3d_5/2_, Mo^6+^ 3d_5/2,_ Mo^4+^ 3d_3/2,_ and Mo^6+^ 3d_3/2,_ respectively, as represented in Fig. 4 (f), [81,82].

### 3.1 Electrochemical study

**Fig. 5:**
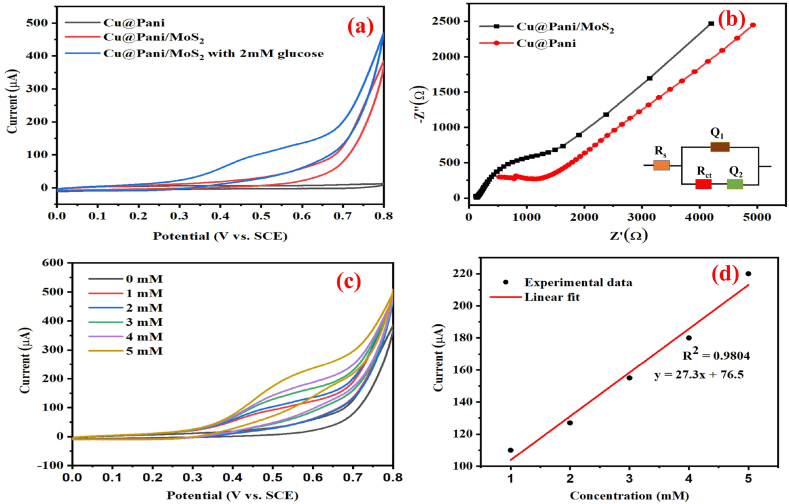
(a) Cyclic voltammograms (CVs) of Cu@Pani and Cu@Pani/MoS_2_ and Cu@Pani/MoS_2_ in the presence of glucose in 0.1 M NaOH at 50 mVs^−1^ (b) Nyquist plot of Cu@Pani and Cu@Pani/MoS_2_ in 5 mM [Fe(CN)_6_]^3-/4-^ redox electrolyte and 0.1 M KCl solution, (c) CV of Cu@Pani/MoS_2_ with different glucose concentrations in the 0.1 M NaOH solution, and (d) Plot of oxidation peak current vs the concentration of glucose solution.

The electrochemical properties of the Cu@Pani, and Cu@Pani/MoS_2_ electrodes were analyzed using 0.1 M NaOH solution as working electrolyte before fabricating a modified electrode for glucose sensing. As shown in Fig. 5(a), using the 0.1 M NaOH electrolyte cyclic voltammogram of Cu@Pani, Cu@Pani/MoS_2_ and Cu@Pani/MoS_2_ in the presence of 2 mM glucose were measured using a three-electrode system [50]. The Cu@Pani electrode exhibited small current response, whereas the current response of the Cu@Pani/MoS_2_ electrode was higher than that of the Cu@Pani electrode. In the presence of 2 mM glucose, the Cu@Pani/MoS_2_ electrode demonstrated a significantly increased current response, which can be attributed to glucose oxidation [14]. The comparatively lower current response of the Cu@Pani can be attributed to the selection of H_2_O_2_ for polymerization instead of ammonium persulfate (APS), and simultaneous use of oxidizing agent (H_2_O_2_) and reducing agent (NaCt), which leads to the incomplete oxidation of aniline to polyaniline, is also represented in Fig. 5(a) [83,84]. The Cu@Pani/MoS_2_ CV spectrum exhibits a current response increment compared to that of Cu@Pani, which can be attributed to the two - dimensional layered structure of MoS_2_ providing a large surface area for electron dispersion at the electrode surface. This greatly increases the electrocatalytic performance of the material [85].

An electrolyte containing a mixture of 5 mM [Fe (CN)_6_]^3−/4−^redox electrolyte and 0.1 M KCl as the supporting electrolyte, was employed to conduct EIS analysis for the further electrochemical study of the Cu@Pani, and Cu@Pani/MoS_2_ electrodes, as revealed in Fig. 5(b). The EIS analysis revealed a semicircular region diameter showing charge transfer resistance (Rct); shifting of the inclined line in the low-frequency region indicated an increased charge transfer kinetics, and diffusion control process respectively for the Cu@Pani/MoS_2_ electrode as compared to that for Cu@Pani electrode [86]. The inset image on Fig. 5(b) shows Randels equivalent circuit, which describes the electrolyte solution resistance (R_s_), R_ct_, double-layer capacitance (Q_1_), and Warburg impedance (Q_2_), where the electrolyte resistance R_s_, is fitted in series combination, and double layer capacitance (Q_1_), R_ct_, and impedance of the faradaic reaction are arranged in parallel combination [[87], [88], [89]].

Fig. 5(c) shows the CV of Cu@Pani and Cu@Pani/MoS_2_ when exposed to 0, 1, 2, 3, 4, and 5 mM glucose solutions. Fig. 5(d) displays a plot of glucose concentration (mM) and anodic current (Ip). A linear regression line with Ip (μA) = 27.3x + 76.5 (R^2^ = 0.9804), reveals that a diffusion-controlled reaction occurred on the Cu@Pani/MoS_2_ electrode surface [90,91]. MoS_2_ nano structure improves the electrocatalytic activity because of its high surface area, electron transfer abilities and rapid diffusion of the electrons and ions on the electrode surface, providing active sites for the electrocatalytic reactions during electrochemical glucose oxidation [92]. The electro-oxidation of glucose in the Cu@Pani/MoS_2_ electrode involves the following reaction mechanisms [93].

(1) Cu + 2OH^−^ → CuO + H_2_O + 2e^−^

(2) CuO + OH^−^ → CuOOH + e^−^

(3) Cu^3+^ + Glucose → Gluconolactone + Cu^2+^

In an alkaline solution, glucose undergoes deprotonation which further initiates the isomerization, thereby forming an enediol structure for further oxidation. In the presence of Cu^3+^, it yields gluconolactone, which produces gluconic acid via hydrolysis [14,94]. The increased oxidation current response after the glucose addition can be attributed to the oxidation of Cu^2+^ to Cu^3+^, which greatly enhances the electrocatalytic activity and electron transfer process at the surface of the electrode material [95,96].

### 3.2 Chronoamperometry analysis

**Fig. 6:**
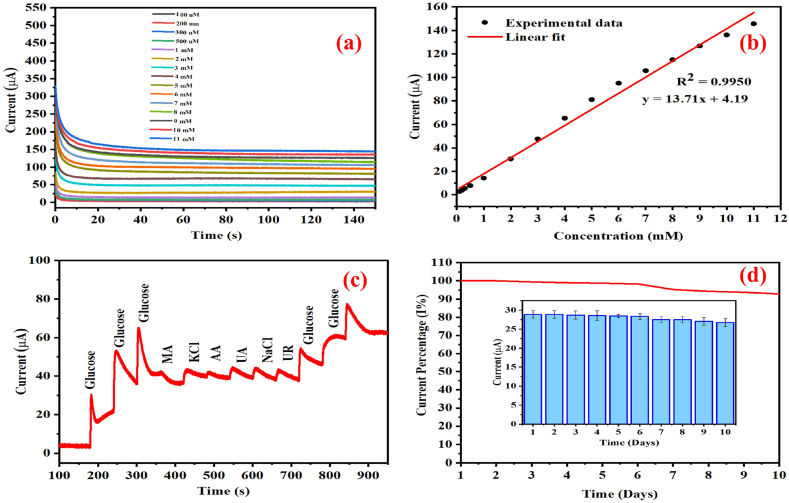
(a) Chronoamperometric i-t curves of the Cu@Pani/MoS_2_ electrode at potential +0.5 V (vs. Hg_2_Cl_2_) at different glucose concentrations (100 μM–11 mM) in 0.1 M NaOH, (b) Plot of current vs. concentration Cu@Pani/MoS_2_, (c) Chronoamperometric response of Cu@Pani/MoS_2_ in 1 mM glucose and 0.1 mM various interfering species in 0.1 M NaOH, (d) Stability of Cu@Pani/MoS_2_ in 2.0 mM glucose solution tested over ten days.

The results of the chronoamperometry analysis of the Cu@Pani/MoS_2_ electrode with glucose are presented in Fig. 6(a). The gradual addition of different glucose concentrations to the Cu@Pani/MoS_2_ electrode led to a gradual increase in the current response, which demonstrate the applicability of the Cu@Pani/MoS_2_ electrode for measuring different glucose concentration. The plot of the observed oxidation current against the glucose concentration, follows a linear regression line with a co-relation coefficient of R^2^ = 0.9950, as illustrated in Fig. 6(b). The linear fitting sensitivity of the electrochemical sensor was calculated and was found to be 69.82 μAmM^−1^cm^−2^. The calculated limit of detection (LOD) was found to be 1.78 μM, which was obtained using the formula LOD = 3.3 σ/m, (where m = slope and σ = standard deviation) [3]. The glucose sensor demonstrated a linear detection range of 100 μM to 11 mM. The obtained results were compared with those of Cu-, polyaniline-, and MoS_2_-based electrode materials available in the literatures, as detailed in Table 1.

**Table 1:** Comparison of polyaniline, Cu, and MoS_2_ based electrodes for the glucose sensor.

Electrode material	Sensitivity μAmM^−1^cm^−2^	Detection limit	Linear detection range	Ref.
CuO-PANI/FTO	1359	0.24 μM	0.28–4.6 mM	[49]
CuO/NiO/PANI/GCE	–	2 μM	0.02–2.5 mM	[97]
PANI-CuNi	1030	0.2 μM	Up to 5.6 mM	[33]
Cu-MWCNTs	1096	1.0 μM	Up to 7.5 mM	[98]
CuOG-SPCE	2367	34.3 nM	0.122 μM- 0.5 mM	[99]
NiNPs-MWCNTs/Copper	3822	0.7 μM	2 μM-10 mM	[100]
Ni–MoS_2_ hybrid	1824	0.31 μM	0–4 mM	[101]
NiCo_2_O_4_@PANI	4550	4.7350 μM	0.3833 mM	[47]
Cu–PANI/ITO	4140	5 μM	0.02–1 mM	[6]
CuO/MoS_2_	1055	0.017 μM	35–800 μM	[102]
Cu/PANI	61.6	9.36 μM	0.02–10 mM	[50]
Cu–MoS_2_	1055	–	0–4 mM	[103]
CuNPs/PANI/graphene	150	0.27 μM	0.001–3.7 mM	[14]
Cu@Pani/MoS_2_	69.82	1.78 μM	0.1–11 mM	Our work

### 3.3 Selectivity, stability, and real sample analysis of the sensor

We further analyzed the selectivity of the Cu@Pani/MoS_2_ electrode by adding 1 mM same volume of glucose solution and different interfering biomolecules such as, maelic acid (MA), potassium chloride (KCl), ascorbic acid (AA), urea (UR), uric acid (UA), and sodium chloride (NaCl) (0.1 mM) [3,104], [105]. Fig. 6(c) shows the selectivity of the Cu@Pani/MoS_2_-based electrochemical non-enzymatic glucose sensor. Our results indicate that there is no noticeable current for interfering biomolecules; but increased current response for glucose, both before and after the addition of interfering biomolecules [106]. The relatively high current response for glucose compared to that of the interfering species, demonstrates the better selectivity of the glucose sensor [49].

Successive chronoamperometric measurements are performed over ten days using 2 mM glucose solution to confirm the stability of the electrode material; the corresponding results are compared in Fig. 6 (d). The results revealed that the sensitivity of the Cu@Pani/MoS_2_ based electrochemical non-enzymatic glucose sensor decreases by 7.25 % and it retains a stability of 92.75 % as depicted in Fig. 6 (d). These results confirm the satisfactory stability of the Cu@Pani/MoS_2_-based sensor. For practical application glucose concentration in human serum (H4522, Sigma - Aldrich, 4.89 mM) sample was measured using the Cu@Pani/MoS_2_-based electrode [3]. For the glucose concentration measurement 20 μL of the serum sample was diluted by adding 30 mL 0.1 M NaOH solution, and the current response was measured using chronoamperometry at 0.5 V potential [33]. Subsequently, different glucose solution of known concentrations (500 μM, 1000 μM, and 2000 μM) were added to the diluted serum solution and current response were recorded using the standard addition method. The corresponding results are displayed in Table 2. Recovery percentages of 94.28 %, 94.92 % and 96.21 % were obtained by adding 500 μM, 1000 μM, and 2000 μM glucose, respectively [95]. Our results demonstrate the viability of the Cu@Pani/MoS_2_ electrode for electrochemical non-enzymatic glucose sensing of biological samples in the presence of interfering species [[107], [108], [109]].

**Table 2:** Recovery test in human blood serum sample.

Samples	Added glucose concentration (μM)	Observed glucose concentration (μM)	Recovery (%)	RSD (%) (n = 3)
1	500	471	94.28	2.15
2	1000	949	94.92	2.08
3	2000	1924	96.21	1.16

## 4 Conclusions

A conclusion, combination of in-situ oxidative polymerization and a hydrothermal process was employed to synthesize Cu@Pani/MoS_2_ nanocomposite. The structural morphology of the nanocomposite was studied using different analytical tools, and the fabricated nanocomposite was used as a non-enzymatic electrochemical glucose sensor. Cu@Pani/MoS_2_ composite-based sensor revealed a sensitivity of 69.82 μAmM^−1^cm^−2^, detection limit of 1.78 μM, and linear range of 100 μM to 11 mM with 92.75 % stability. The Cu@Pani/MoS_2_ nanocomposite exhibited effective sensitivity, good selectivity, stability, and a short electrochemical response time, which indicates that the Cu@Pani/MoS_2_ composite electrode is viable for electrochemical non-enzymatic glucose sensor. Our work provides a new method for the design of composite electrode materials for electrochemical applications such as supercapacitor, electrochemical sensor, batteries etc.

## Data availability statement

Data will be made available on request.

## CRediT authorship contribution statement

**Krishna Prasad Sharma:** Conceptualization, Data curation, Formal analysis, Investigation, Methodology, Visualization, Writing - original draft, Writing - review & editing. **Miyeon Shin:** Data curation, Formal analysis, Project administration, Resources, Software, Writing – review & editing. **Kyong Kim:** Data curation, Project administration, Resources, Software. **Kyungmin Woo:** Data curation, Formal analysis, Investigation, Resources, Software. **Ganesh Prasad Awasthi:** Data curation, Formal analysis, Methodology, Validation. **Changho Yu:** Formal analysis, Funding acquisition, Project administration, Resources, Supervision, Validation, Writing – review & editing.

## Declaration of competing interest

There is no conflict of interest to disclose for this research paper. The human serum samples were not taken form human patients. We have used commercially available human serum (H4522, Sigma- Aldrich) for the real sample analysis.

## References

[1] Fang L., Wang F., Chen Z., Qiu Y., Zhai T., Hu M., Zhang C., Huang K. (2017). Flower-like MoS2 decorated with Cu2O nanoparticles for non-enzymatic amperometric sensing of glucose. Talanta.

[2] Alberti K.G., Zimmet P.Z. (1998). Definition, diagnosis and classification of diabetes mellitus and its complications. Part 1: diagnosis and classification of diabetes mellitus provisional report of a WHO consultation. Diabet. Med. : a journal of the British Diabetic Association.

[3] Sharma K.P., Shin M., Awasthi G.P., Poudel M.B., Kim H.J., Yu C. (2022). Chitosan polymer matrix-derived nanocomposite (CuS/NSC) for non-enzymatic electrochemical glucose sensor. Int. J. Biol. Macromol..

[4] G.J.I.J.o.N.D. Roglic (2016). WHO Global report on diabetes. A summary.

[5] Evtugyn G., Porfireva A., Hianik T., Tiwari A., Patra H.K., Turner A.P.F. (2015). Electropolymerized Materials for Biosensors, Advanced Bioelectronics Materials.

[6] Belgherbi O., Chouder D., Lakhdari D., Dehchar C., Laidoudi S., Lamiri L., Hamam A., Seid L. (2020). Enzyme-free glucose sensor based on Star-like copper particles-polyaniline composite film. J. Inorg. Organomet. Polym. Mater..

[7] Baghayeri M., Nodehi M., Amiri A., Amirzadeh N., Behazin R., Iqbal M.Z. (2020). Electrode designed with a nanocomposite film of CuO Honeycombs/Ag nanoparticles electrogenerated on a magnetic platform as an amperometric glucose sensor. Anal. Chim. Acta.

[8] Baghayeri M., Amiri A., Motamedifar A. (2016). Investigation about electrocatalytic oxidation of glucose on loaded Ag nanoparticles on functionalized carbon nanotubes. Ionics.

[9] Baghayeri M., Amiri A., Alizadeh Z., Veisi H., Hasheminejad E. (2018). Non-enzymatic voltammetric glucose sensor made of ternary NiO/Fe3O4-SH/para-amino hippuric acid nanocomposite. J. Electroanal. Chem..

[10] Cash K.J., Clark H.A. (2010). Nanosensors and nanomaterials for monitoring glucose in diabetes. Trends Mol. Med..

[11] Vafapour Z. (2019). Polarization-independent perfect optical metamaterial absorber as a glucose sensor in food Industry applications. IEEE Trans. NanoBioscience.

[12] Pullano S.A., Greco M., Bianco M.G., Foti D., Brunetti A., Fiorillo A.S. (2022). Glucose biosensors in clinical practice: principles, limits and perspectives of currently used devices. Theranostics.

[13] Huang K.-J., Wang L., Li J., Gan T., Liu Y.-M. (2013). Glassy carbon electrode modified with glucose oxidase–graphene–nano-copper composite film for glucose sensing. Measurement.

[14] Zheng W., Hu L., Lee L.Y.S., Wong K.-Y. (2016). Copper nanoparticles/polyaniline/graphene composite as a highly sensitive electrochemical glucose sensor. J. Electroanal. Chem..

[15] Hou X., Xu H., Zhen T., Wu W. (2020). Recent developments in three-dimensional graphene-based electrochemical sensors for food analysis. Trends Food Sci. Technol..

[16] Chiu W.-T., Chang T.-F.M., Sone M., Hosoda H., Tixier-Mita A., Toshiyoshi H. (2021).

[17] Qu K., Wang S., He W., Yin H., Wang L., Zheng Y. (2023). Ternary metal oxide nanorods (Ni0.5Cu0.5Co2O4) as efficient positive materials for non-enzymatic glucose sensing and fuel cell application. Solid State Sci..

[18] Yang Q., Zhang X., Kumar S., Singh R., Zhang B., Bai C., Pu X.J.P. (2020). Development of glucose sensor using gold nanoparticles and glucose-oxidase functionalized tapered fiber structure.

[19] Baghayeri M., Amiri A., Farhadi S. (2016). Development of non-enzymatic glucose sensor based on efficient loading Ag nanoparticles on functionalized carbon nanotubes. Sensor. Actuator. B Chem..

[20] Abdul Amir Al-Mokaram A.M.A., Yahya R., Abdi M.M., Muhammad Ekramul Mahmud H.N. (2016). One-step electrochemical deposition of Polypyrrole–Chitosan–Iron oxide nanocomposite films for non-enzymatic glucose biosensor. Mater. Lett..

[21] Guan J.-F., Huang Z.-N., Zou J., Jiang X.-Y., Peng D.-M., Yu J.-G. (2020). A sensitive non-enzymatic electrochemical sensor based on acicular manganese dioxide modified graphene nanosheets composite for hydrogen peroxide detection. Ecotoxicol. Environ. Saf..

[22] Qu K., Wang S., He W., Yin H., Zhao S., Wang L., Zheng Y. (2023). One-pot self-assembled two-dimensional Ni/Ni3C/C3N4 nanosheets with highly efficient glucose oxidation for fuel cell and sensing applications. Mater. Today Commun..

[23] Vinoth V., Subramaniyam G., Anandan S., Valdés H., Manidurai P. (2021). Non-enzymatic glucose sensor and photocurrent performance of zinc oxide quantum dots supported multi-walled carbon nanotubes. Mater. Sci. Eng., B.

[24] Khalaf N., Ahamad T., Naushad M., Al-Hokbany N., Al-Saeedi S.I., Almotairi S., Alshehri S.M. (2020). Chitosan polymer complex derived nanocomposite (AgNPs/NSC) for electrochemical non-enzymatic glucose sensor. Int. J. Biol. Macromol..

[25] Chen J., Yin H., Zhou J., Wang L., Gong J., Ji Z., Nie Q. (2020). Efficient nonenzymatic sensors based on Ni-mof microspheres decorated with Au nanoparticles for glucose detection. J. Electron. Mater..

[26] Zhou J., Yin H., Chen J., Gong J., Wang L., Zheng Y., Nie Q. (2020). Electrodeposition of bimetallic NiAu alloy dendrites on carbon papers as highly sensitive disposable non-enzymatic glucose sensors. Mater. Lett..

[27] Dakshayini B.S., Reddy K.R., Mishra A., Shetti N.P., Malode S.J., Basu S., Naveen S., Raghu A.V. (2019). Role of conducting polymer and metal oxide-based hybrids for applications in ampereometric sensors and biosensors. Microchem. J..

[28] Karimi-Maleh H., Liu Y., Li Z., Darabi R., Orooji Y., Karaman C., Karimi F., Baghayeri M., Rouhi J., Fu L., Rostamnia S., Rajendran S., Sanati A.L., Sadeghifar H., Ghalkhani M. (2023). Calf thymus ds-DNA intercalation with pendimethalin herbicide at the surface of ZIF-8/Co/rGO/C3N4/ds-DNA/SPCE; A bio-sensing approach for pendimethalin quantification confirmed by molecular docking study. Chemosphere.

[29] Ermis N., Zare N., Darabi R., Alizadeh M., Karimi F., Singh J., Shahidi S.-A., Dragoi E.N., Camarada M.B., Baghayeri M. (2023). Recent advantage in electrochemical monitoring of gallic acid and kojic acid: a new perspective in food science. J. Food Meas. Char..

[30] Karimi-Maleh H., Darabi R., Karimi F., Karaman C., Shahidi S.A., Zare N., Baghayeri M., Fu L., Rostamnia S., Rouhi J., Rajendran S. (2023). State-of-art advances on removal, degradation and electrochemical monitoring of 4-aminophenol pollutants in real samples: a review. Environ. Res..

[31] Liu H., Baghayeri M., Amiri A., Karimabadi F., Nodehi M., Fayazi M., Maleki B., Zare‬ E.N., Kaffash A. (2023). A strategy for As(III) determination based on ultrafine gold nanoparticles decorated on magnetic graphene oxide. Environ. Res..

[32] Wang Y., Liu A., Han Y., Li T. (2019). Sensors based on conductive polymers and their composites: a review. Polym. Int..

[33] Bilal S., Ullah W., Ali Shah A.-u.-H. (2018). Polyaniline@CuNi nanocomposite: a highly selective, stable and efficient electrode material for binder free non-enzymatic glucose sensor. Electrochim. Acta.

[34] Liu Y., Tang D., Cao K., Yu L., Han J., Xu Q. (2018). Probing the support effect at the molecular level in the polyaniline-supported palladium nanoparticle-catalyzed Ullmann reaction of aryl iodides. J. Catal..

[35] Sultana S., Rafiuddin, Zain Khan M., Umar K. (2012). Synthesis and characterization of copper ferrite nanoparticles doped polyaniline. J. Alloys Compd..

[36] Kang E.T., Neoh K.G., Tan K.L. (1998). Polyaniline: a polymer with many interesting intrinsic redox states. Prog. Polym. Sci..

[37] Huang K.-J., Wang L., Liu Y.-J., Wang H.-B., Liu Y.-M., Wang L.-L. (2013). Synthesis of polyaniline/2-dimensional graphene analog MoS2 composites for high-performance supercapacitor. Electrochim. Acta.

[38] Divya V., Sangaranarayanan M.V. (2012). A facile synthetic strategy for mesoporous crystalline copper–polyaniline composite. Eur. Polym. J..

[39] Ramakrishnan S., Balamurugan J., Vinothkannan M., Kim A.R., Sengodan S., Yoo D.J. (2020). Nitrogen-doped graphene encapsulated FeCoMoS nanoparticles as advanced trifunctional catalyst for water splitting devices and zinc–air batteries. Appl. Catal. B Environ..

[40] Wu M.-h., Li L., Liu N., Wang D.-j., Xue Y.-c., Tang L. (2018). Molybdenum disulfide (MoS 2) as a co-catalyst for photocatalytic degradation of organic contaminants: a review. Process Saf. Environ. Protect..

[41] Raghu M.S., Kumar K.Y., Rao S., Aravinda T., Prasanna B.P., Prashanth M.K. (2018). Fabrication of polyaniline–few-layer MoS2 nanocomposite for high energy density supercapacitors. Polym. Bull..

[42] Poudel M., Kim A. (2021). Direct growth of MoS 2 -Ni 3 S 2 core-shell nanowire array on nickel foam as a bifunctional electrode for supercapacitor and electro-oxidation of methanol. Chalcogenide Lett..

[43] Xu F., Wu M., Ma G., Xu H., Shang W. (2020). Copper-molybdenum sulfide/reduced graphene oxide hybrid with three-dimensional wrinkles and pores for enhanced amperometric detection of glucose. Microchem. J..

[44] He W., Huang Y., Wu J. (2020). Enzyme-free glucose biosensors based on MoS2 nanocomposites. Nanoscale Res. Lett..

[45] Gautam K.P., Acharya D., Bhatta I., Subedi V., Das M., Neupane S., Kunwar J., Chhetri K., Yadav A.P. (2022). Nickel oxide-incorporated polyaniline nanocomposites as an efficient electrode material for supercapacitor application. Inorganics.

[46] Awasthi G.P., Poudel M.B., Shin M., Sharma K.P., Kim H.J., Yu C. (2021). Facile synthesis of a copper oxide/molybdenum disulfide heterostructure for asymmetric supercapacitors of high specific energy. J. Energy Storage.

[47] Yu Z., Li H., Zhang X., Liu N., Tan W., Zhang X., Zhang L. (2016). Facile synthesis of NiCo2O4@Polyaniline core–shell nanocomposite for sensitive determination of glucose. Biosens. Bioelectron..

[48] Yang J., Zhang W.D., Gunasekaran S. (2010). An amperometric non-enzymatic glucose sensor by electrodepositing copper nanocubes onto vertically well-aligned multi-walled carbon nanotube arrays. Biosens. Bioelectron..

[49] Esmaeeli A., Ghaffarinejad A., Zahedi A., Vahidi O. (2018). Copper oxide-polyaniline nanofiber modified fluorine doped tin oxide (FTO) electrode as non-enzymatic glucose sensor. Sensor. Actuator. B Chem..

[50] Prathap M.U.A., Pandiyan T., Srivastava R. (2013). Cu nanoparticles supported mesoporous polyaniline and its applications towards non-enzymatic sensing of glucose and electrocatalytic oxidation of methanol. J. Polym. Res..

[51] Chen Y., Zhang Q., Jing X., Han J., Yu L. (2019). Synthesis of Cu-doped polyaniline nanocomposites (nano Cu@PANI) via the H2O2-promoted oxidative polymerization of aniline with copper salt. Mater. Lett..

[52] Shomalian K., Bagheri-Mohagheghi M.M., Ardyanian M. (2016). Characterization and study of reduction and sulfurization processing in phase transition from molybdenum oxide (MoO2) to molybdenum disulfide (MoS2) chalcogenide semiconductor nanoparticles prepared by one-stage chemical reduction method. Appl. Phys. A.

[53] Kishore P., Viswanathan B., Varadarajan T. (2008). Synthesis and characterization of metal nanoparticle embedded conducting polymer–polyoxometalate composites. Nanoscale Res. Lett..

[54] Millero F.J., Sharma V.K., Karn B. (1991). The rate of reduction of copper(II) with hydrogen peroxide in seawater. Mar. Chem..

[55] Bogdanović U., Dimitrijević S., Škapin S.D., Popović M., Rakočević Z., Leskovac A., Petrović S., Stoiljković M., Vodnik V. (2018). Copper-polyaniline nanocomposite: role of physicochemical properties on the antimicrobial activity and genotoxicity evaluation. Mater. Sci. Eng. C.

[56] Tyagi H., Kushwaha A., Kumar A., Aslam M. (2016). A facile pH controlled citrate-based reduction method for gold nanoparticle synthesis at room temperature. Nanoscale Res. Lett..

[57] Chen X., Meng F., Zhou Z., Tian X., Shan L., Zhu S., Xu X., Jiang M., Wang L., Hui D., Wang Y., Lu J., Gou J. (2014). One-step synthesis of graphene/polyaniline hybrids by in situ intercalation polymerization and their electromagnetic properties. Nanoscale.

[58] Wang R.-X., Huang L.-F., Tian X.-Y. (2012). Understanding the protonation of polyaniline and polyaniline–graphene interaction. J. Phys. Chem. C.

[59] Xu H., Zhang J., Chen Y., Lu H., Zhuang J. (2014). Electrochemical polymerization of polyaniline doped with Cu2+ as the electrode material for electrochemical supercapacitors. RSC Adv..

[60] Bhattarai D.P., Hwang T.I., Kim J.I., Lee J.H., Chun S., Kim B.-S., Park C.H., Kim C.S. (2020). Synthesis of polypyrrole nanorods via sacrificial removal of aluminum oxide nanopore template: a study on cell viability, electrical stimulation and neuronal differentiation of PC12 cells. Mater. Sci. Eng. C.

[61] Masud R., Islam M.S., Haque P., Khan M., Shahruzzaman M., Khan M., Takafuji M., Rahman M. (2020). Preparation of novel chitosan/poly (ethylene glycol)/ZnO bionanocomposite for wound healing application: effect of gentamicin loading. Materialia.

[62] Liu N., Cheng J., Hou W., Yang C., Yang X., Zhou J. (2022). Bottom-up synthesis of two-dimensional composite via CuBDC-ns growth on multilayered MoS2 to boost CO2 permeability and selectivity in Pebax-based mixed matrix membranes. Separation and Purification Technology.

[63] Wang L.-C., Bao S.-K., Luo J., Wang Y.-H., Nie Y.-C., Zou J.-P. (2016). Efficient exfoliation of bulk MoS2 to nanosheets by mixed-solvent refluxing method. Int. J. Hydrogen Energy.

[64] Fuseini M., El-Shazly A.H., El-Kady M.F. (2020). Effects of doping on zeta potential and pH of polyaniline colloidal Suspension. Mater. Sci. Forum.

[65] Fu Y., An Q., Ni R., Zhang Y., Li Y., Ke H. (2018). Preparation of polyaniline-encapsulated carbon/copper composite nanofibers for detection of polyphenol pollutant. Colloids Surf. A Physicochem. Eng. Asp..

[66] Palsaniya S., Nemade H.B., Dasmahapatra A.K. (2018). Synthesis of polyaniline/graphene/MoS2 nanocomposite for high performance supercapacitor electrode. Polymer.

[67] Bai J., Zhao B., Wang X., Ma H., Li K., Fang Z., Li H., Dai J., Zhu X., Sun Y. (2020). Yarn ball-like MoS2 nanospheres coated by nitrogen-doped carbon for enhanced lithium and sodium storage performance. J. Power Sources.

[68] Žalnėravičius R., Klimas V., Paškevičius A., Grincienė G., Karpicz R., Jagminas A., Ramanavičius A. (2021). Highly efficient antimicrobial agents based on sulfur-enriched, hydrophilic molybdenum disulfide nano/microparticles and coatings functionalized with palladium nanoparticles. J. Colloid Interface Sci..

[69] Gao Y., Chen C., Tan X., Xu H., Zhu K. (2016). Polyaniline-modified 3D-flower-like molybdenum disulfide composite for efficient adsorption/photocatalytic reduction of Cr(VI). J. Colloid Interface Sci..

[70] Zhang X., Ma L., Gan M., Fu G., Jin M., Zhai Y. (2018). Controllable constructing of hollow MoS2/PANI core/shell microsphere for energy storage. Appl. Surf. Sci..

[71] Adhikari S., Sarkar D., Madras G. (2017). Hierarchical design of CuS architectures for visible light photocatalysis of 4-chlorophenol. ACS Omega.

[72] Prasad Ojha G., Muthurasu A., Prasad Tiwari A., Pant B., Chhetri K., Mukhiya T., Dahal B., Lee M., Park M., Kim H.-Y. (2020). Vapor solid phase grown hierarchical CuxO NWs integrated MOFs-derived CoS2 electrode for high-performance asymmetric supercapacitors and the oxygen evolution reaction. Chem. Eng. J..

[73] Huan K., Li Y., Deng D., Wang H., Wang D., Li M., Luo L. (2022). Composite-controlled electrospinning of CuSn bimetallic nanoparticles/carbon nanofibers for electrochemical glucose sensor. Appl. Surf. Sci..

[74] Acharya J., Pant B., Prasad Ojha G., Park M. (2022). Embellishing hierarchical 3D core-shell nanosheet arrays of ZnFe2O4@NiMoO4 onto rGO-Ni foam as a binder-free electrode for asymmetric supercapacitors with excellent electrochemical performance. J. Colloid Interface Sci..

[75] Lohani P.C., Tiwari A.P., Chhetri K., Muthurasu A., Dahal B., Chae S.-H., Ko T.H., Lee J.Y., Chung Y.S., Kim H.Y. (2022). Polypyrrole nanotunnels with luminal and abluminal layered double hydroxide nanosheets grown on a carbon cloth for energy storage applications. ACS Appl. Mater. Interfaces.

[76] Munir A., Ulhaq T., Qurashi A., Ur Rehman H., Ul-Hamid A., Hussain I. (2018). Ultrasmall Ni/NiO nanoclusters on thiol functionalized and exfoliated graphene oxide nanosheets for durable oxygen evolution reaction. ACS Appl. Energy Mater..

[77] Kandel D.R., Kim H.-J., Lim J.-M., Poudel M.B., Cho M., Kim H.-W., Oh B.-T., Nah C., Lee S.H., Dahal B., Lee J. (2022). Cold plasma-assisted regeneration of biochar for dye adsorption. Chemosphere.

[78] Dong H., Liu C., Ye H., Hu L., Fugetsu B., Dai W., Cao Y., Qi X., Lu H., Zhang X. (2015). Three-dimensional nitrogen-doped graphene supported molybdenum disulfide nanoparticles as an advanced catalyst for hydrogen evolution reaction. Sci. Rep..

[79] Kim T., Subedi S., Dahal B., Chhetri K., Mukhiya T., Muthurasu A., Gautam J., Lohani P.C., Acharya D., Pathak I. (2022). Homogeneous elongation of N‐doped CNTs over nano‐fibrillated hollow‐carbon‐nanofiber: mass and charge balance in asymmetric supercapacitors is No longer problematic. Adv. Sci..

[80] Ghising R.B., Pan U.N., Paudel D.R., Kandel M.R., Kim N.H., Lee J.H. (2022). A hybrid trimetallic–organic framework-derived N, C co-doped Ni–Fe–Mn–P ultrathin nanosheet electrocatalyst for proficient overall water-splitting. J. Mater. Chem. A.

[81] Zou L., Qu R., Gao H., Guan X., Qi X., Liu C., Zhang Z., Lei X. (2019). MoS2/RGO hybrids prepared by a hydrothermal route as a highly efficient catalytic for sonocatalytic degradation of methylene blue. Results Phys..

[82] Kandel M.R., Pan U.N., Paudel D.R., Dhakal P.P., Kim N.H., Lee J.H. (2022). Hybridized bimetallic phosphides of Ni–Mo, Co–Mo, and Co–Ni in a single ultrathin-3D-nanosheets for efficient HER and OER in alkaline media. Compos. B Eng..

[83] Wang Y., Jing X., Kong J. (2007). Polyaniline nanofibers prepared with hydrogen peroxide as oxidant. Synth. Met..

[84] Sapurina I.Y., Shishov M.A. (2012). Oxidative polymerization of aniline: molecular synthesis of polyaniline and the formation of supramolecular structures. New polymers for special applications.

[85] Arunbalaji S., Vasudevan R., Arivanandhan M., Alsalme A., Alghamdi A., Jayavel R. (2020). CuO/MoS2 nanocomposites for rapid and high sensitive non-enzymatic glucose sensors. Ceram. Int..

[86] Lan B., Zhang X., Wang Y., Wei C., Wen G. (2022). Constructing highly stable lithium storage materials by improving the bond strength of MoS2 to graphene via chitosan. Carbon.

[87] Rajaji U., Ganesh P.-S., Chen S.-M., Govindasamy M., Kim S.-Y., Alshgari R.A., Shimoga G. (2021). Deep eutectic solvents synthesis of perovskite type cerium aluminate embedded carbon nitride catalyst: high-sensitive amperometric platform for sensing of glucose in biological fluids. J. Ind. Eng. Chem..

[88] Ramanavicius A., Genys P., Ramanaviciene A. (2014). Electrochemical impedance spectroscopy based evaluation of 1,10-Phenanthroline-5,6-dione and glucose oxidase modified graphite electrode. Electrochim. Acta.

[89] Shrestha B.K., Ahmad R., Shrestha S., Park C.H., Kim C.S. (2017). In situ synthesis of cylindrical spongy polypyrrole doped protonated graphitic carbon nitride for cholesterol sensing application. Biosens. Bioelectron..

[90] Kim S.e., Muthurasu A. (2020). Metal-organic framework–assisted bimetallic Ni@Cu microsphere for enzyme-free electrochemical sensing of glucose. J. Electroanal. Chem..

[91] Jin W., Fu Y., Cai W. (2019). In situ growth of CuS decorated graphene oxide-multiwalled carbon nanotubes for ultrasensitive H2O2 detection in alkaline solution. New J. Chem..

[92] Van Tuan D., Thuy Ngan D.T., Thuy N.T., Lan H., Nguyet N.T., Van Thu V., Hung V.-P., Tam P.D. (2020). Effect of nanostructured MoS2 morphology on the glucose sensing of electrochemical biosensors. Curr. Appl. Phys..

[93] Sharma K.P., Shin M., Awasthi G.P., Yu C. (2023). Single step hydrothermal synthesis of CuS/MnS composite for electrochemical non-enzymatic glucose sensor. Solid State Sci..

[94] Gao P., Zhang Y., Abedi H. (2020). Hierarchical CuS doped with vanadium nanosheets with micro skein overall morphology as a high performance amperometric glucose sensor. Surface. Interfac..

[95] Fu Y., Jin W. (2019). Facile synthesis of core-shell CuS-Cu2S based nanocomposite for the high-performance glucose detection. Mater. Sci. Eng. C.

[96] Opallo M., Dolinska J., Wandelt K. (2018). Encyclopedia of Interfacial Chemistry.

[97] Ghanbari K., Babaei Z. (2016). Fabrication and characterization of non-enzymatic glucose sensor based on ternary NiO/CuO/polyaniline nanocomposite. Anal. Biochem..

[98] Yang J., Zhang W.-D., Gunasekaran S. (2010). An amperometric non-enzymatic glucose sensor by electrodepositing copper nanocubes onto vertically well-aligned multi-walled carbon nanotube arrays. Biosens. Bioelectron..

[99] Sun C.-L., Cheng W.-L., Hsu T.-K., Chang C.-W., Chang J.-L., Zen J.-M. (2013). Ultrasensitive and highly stable nonenzymatic glucose sensor by a CuO/graphene-modified screen-printed carbon electrode integrated with flow-injection analysis. Electrochem. Commun..

[100] Zhong A., Luo X., Chen L., Wei S., Liang Y., Li X. (2015). Enzyme-free sensing of glucose on a copper electrode modified with nickel nanoparticles and multiwalled carbon nanotubes. Microchim. Acta.

[101] Huang J., He Y., Jin J., Li Y., Dong Z., Li R. (2014). A novel glucose sensor based on MoS2 nanosheet functionalized with Ni nanoparticles. Electrochim. Acta.

[102] Arunbalaji S., Vasudevan R., Arivanandhan M., Alsalme A., Alghamdi A., Jayavel R. (2020). CuO/MoS2 nanocomposites for rapid and high sensitive non-enzymatic glucose sensors. Ceram. Int..

[103] Huang J., Dong Z., Li Y., Li J., Tang W., Yang H., Wang J., Bao Y., Jin J., Li R. (2013). MoS2 nanosheet functionalized with Cu nanoparticles and its application for glucose detection. Mater. Res. Bull..

[104] Lu Y., Qiu K., Zhang D., Lin J., Xu J., Liu X., Tang C., Kim J.-K., Luo Y. (2014). Cost-effective CuO nanotube electrodes for energy storage and non-enzymatic glucose detection. RSC Adv..

[105] Emir G., Dilgin Y., Ramanaviciene A., Ramanavicius A. (2021). Amperometric nonenzymatic glucose biosensor based on graphite rod electrode modified by Ni-nanoparticle/polypyrrole composite. Microchem. J..

[106] Omotayo A., Nwahara N., Mwanza D., Nyokong T., Mashazi P. (2023). High-performance non-enzymatic glucose sensing on nanocomposite electrocatalysts of nickel phthalocyanine nanorods and nitrogen doped-reduced graphene oxide nanosheets. Appl. Surf. Sci..

[107] Shrestha B.K., Ahmad R., Mousa H.M., Kim I.-G., Kim J.I., Neupane M.P., Park C.H., Kim C.S. (2016). High-performance glucose biosensor based on chitosan-glucose oxidase immobilized polypyrrole/Nafion/functionalized multi-walled carbon nanotubes bio-nanohybrid film. J. Colloid Interface Sci..

[108] Kim S.e., Muthurasu A.J.E. (2021). Highly oriented nitrogen‐doped carbon nanotube integrated bimetallic cobalt copper organic framework for non‐enzymatic electrochemical glucose and hydrogen peroxide. Sensor.

[109] Gupta P., Gupta V.K., Huseinov A., Rahm C.E., Gazica K., Alvarez N.T. (2021). Highly sensitive non-enzymatic glucose sensor based on carbon nanotube microelectrode set. Sensor. Actuator. B Chem..

