# Supplementary material for: Copper nanoparticles/polyaniline/molybdenum disulfide composite as a nonenzymatic electrochemical glucose sensor
Source: Heliyon. 2023 Oct 26;9(12):e21272. doi: 10.1016/j.heliyon.2023.e21272 (PMC10709213; doi:10.1016/j.heliyon.2023.e21272)
Supplement: Multimedia component 1 [file mmc1.docx]

**Supplementary Materials**

Copper nanoparticles/polyaniline/molybdenum disulfide composite as a nonenzymatic electrochemical glucose sensor

Krishna Prasad Sharma ^a1^, Miyeon Shin^a1^, Kyong Kim ^b^, Kyungmin Woo ^c^, Ganesh Prasad Awasthi ^c^, Changho Yu ^a,c,*^

^a^ Department of Energy Storage/Conversion Engineering (BK21 FOUR), Jeonbuk National University, Jeonju, Jeollabuk-do, 54896, Republic of Korea

^b^ Department of Rehabilitation Engineering, Daegu Hanny University, Gyeongsan, Gyeongsangbuk-do, 38609, Republic of Korea

^c^ Division of Convergence Technology Engineering, Jeonbuk National University, Jeonju, Jeollabuk-do, 54896, Republic of Korea

* To whom correspondence should be addressed.

^†^ These authors contributed equally to this work.

E-mail: goody0418@jbnu.ac.kr, Telephone: +82-63-472-2898, Fax: +82-63-270-4226

**Supporting Information**

1. **Calculation of surface area of the Cu@Pani/MoS_2_ loaded electrode**

The geometric surface area glassy carbon electrode (GCE) was used from formula (A = πr^2^ or πd^2^/4) for the sensitivity calculation. Where r = radius and d = diameter of the glassy carbon electrode. Here, diameter of glassy carbon= 5 mm (Since 1 cm = 10 mm)

Surface area of Glassy carbon electrode = πd^2^/4

= 3.1416*25/4 = 19.635 mm^2^ = 0.19635 cm^2^

Sensitivity and detection limit were calculated by using following formulas,

Sensitivity = Slope/ area

LOD= 3*σ/m, where m= slope from cal. curve, σ = standard deviation.

Based on geometrical surface area of electrode, sensitivity calculation is also reported by Zheng et al, and Gao et al, as cited in the 14 and 69 reference number of our manuscript.


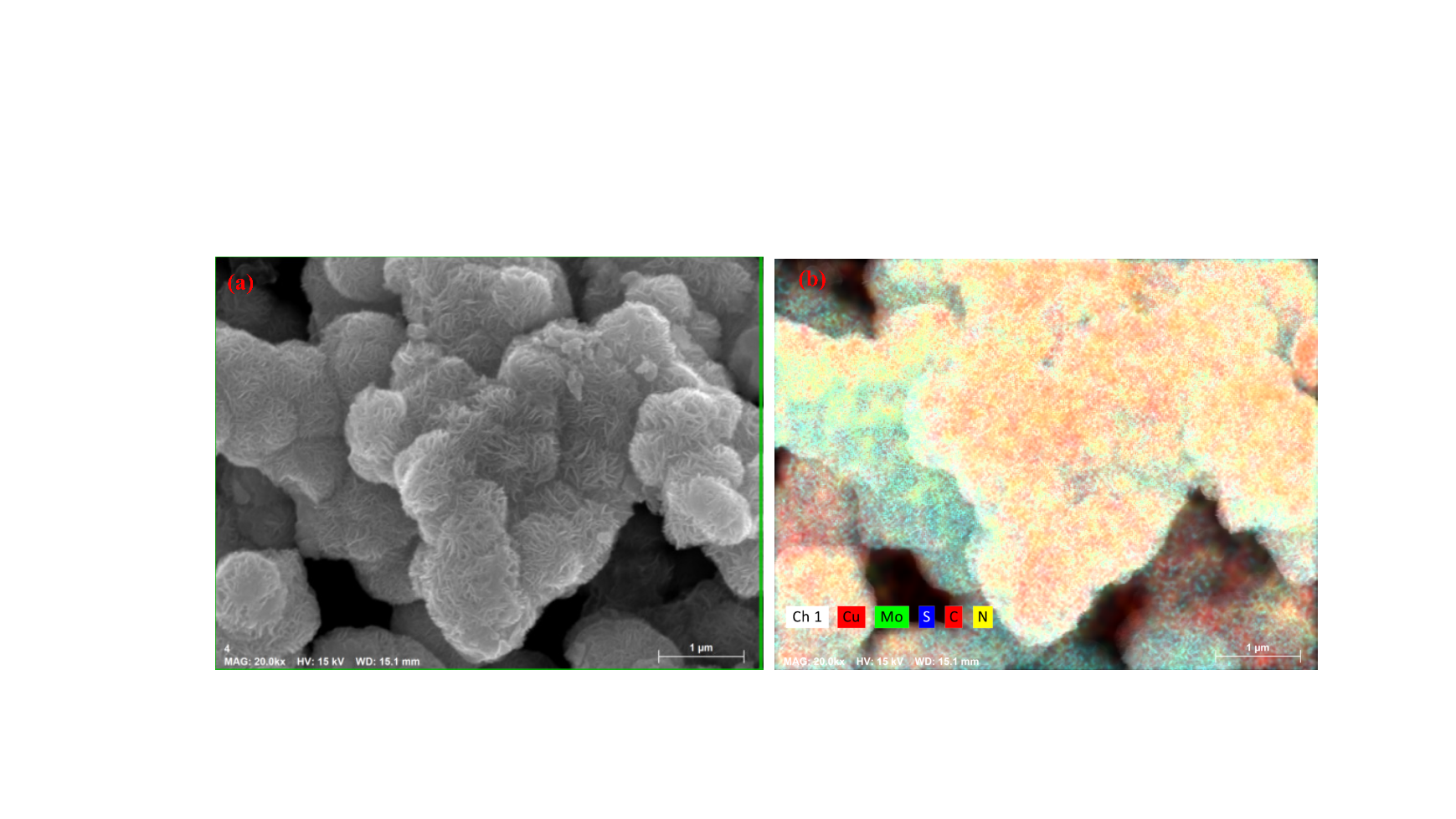


**Fig.S1.** (a) FE-SEM image of Cu@Pani/MoS_2_ (b) EDX mapping of Cu@Pani/MoS_2_.


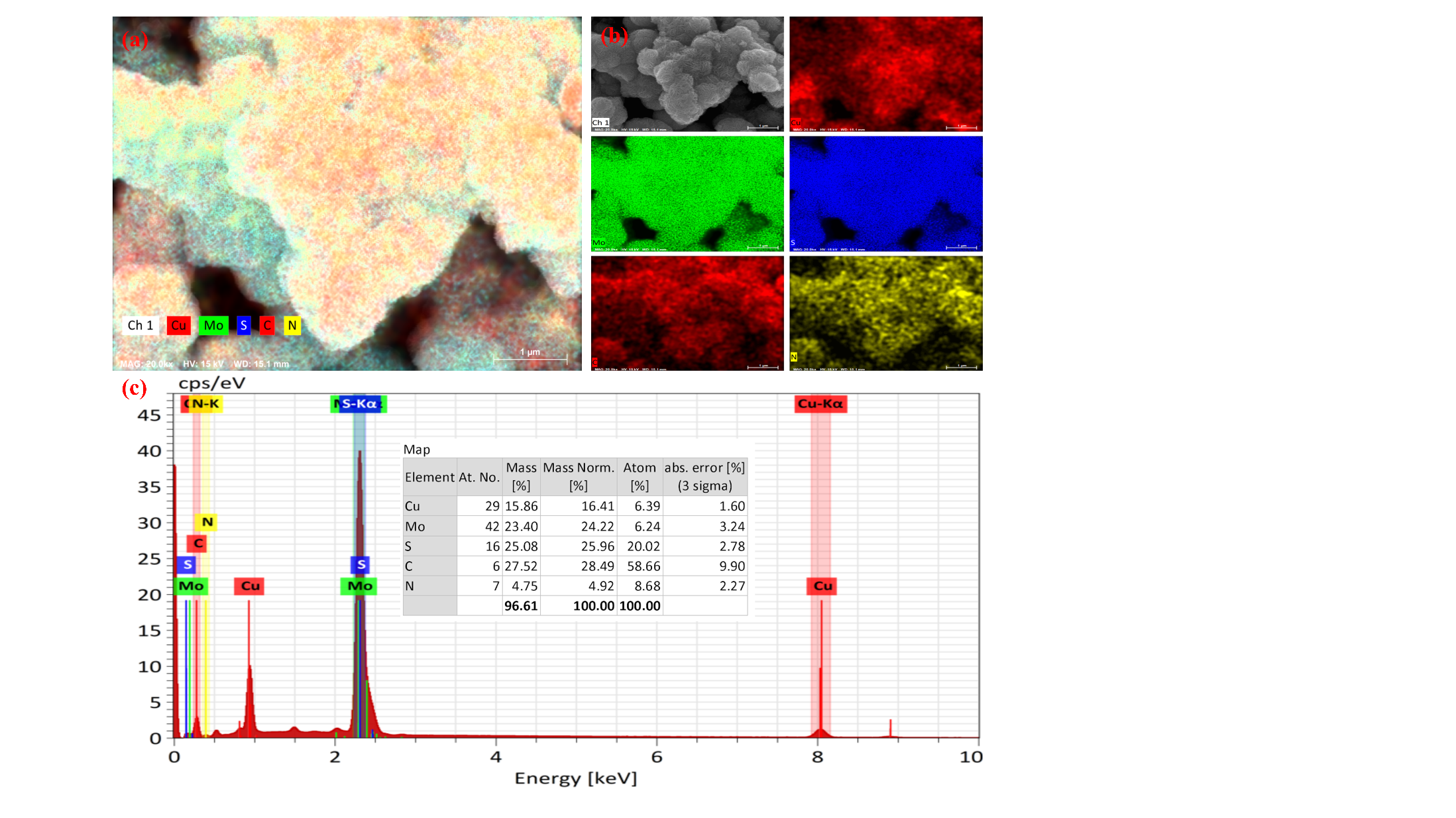


**Fig.S2.** (a, b) EDX mapping of Cu@Pani/MoS_2_ with individual elemental mapping, (c) EDX spectrum with elemental analysis of Cu@Pani/MoS_2_.


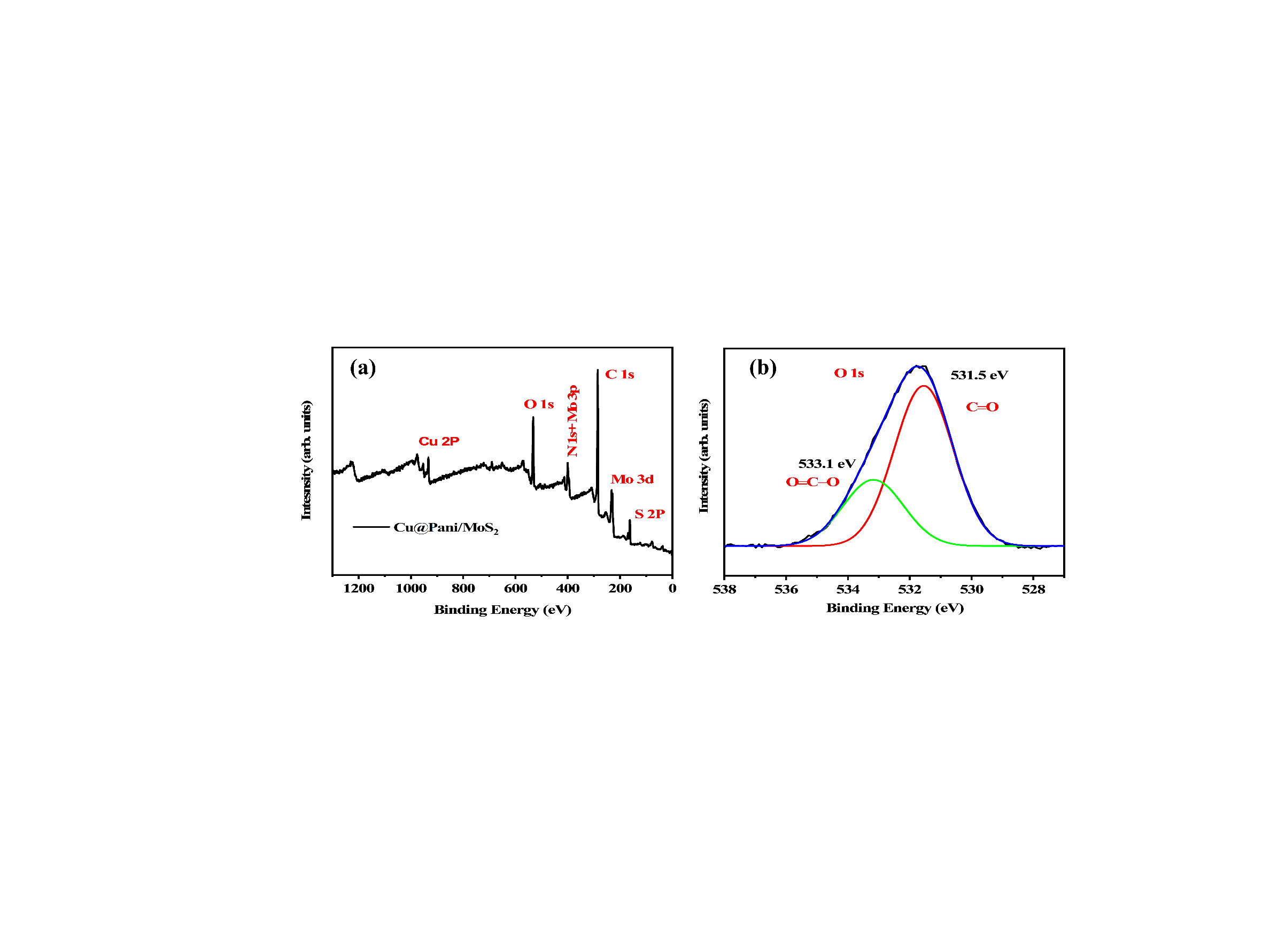


**Fig. S3.** (a) XPS spectra of Cu@Pani/MoS_2_ and (b) O 1s.


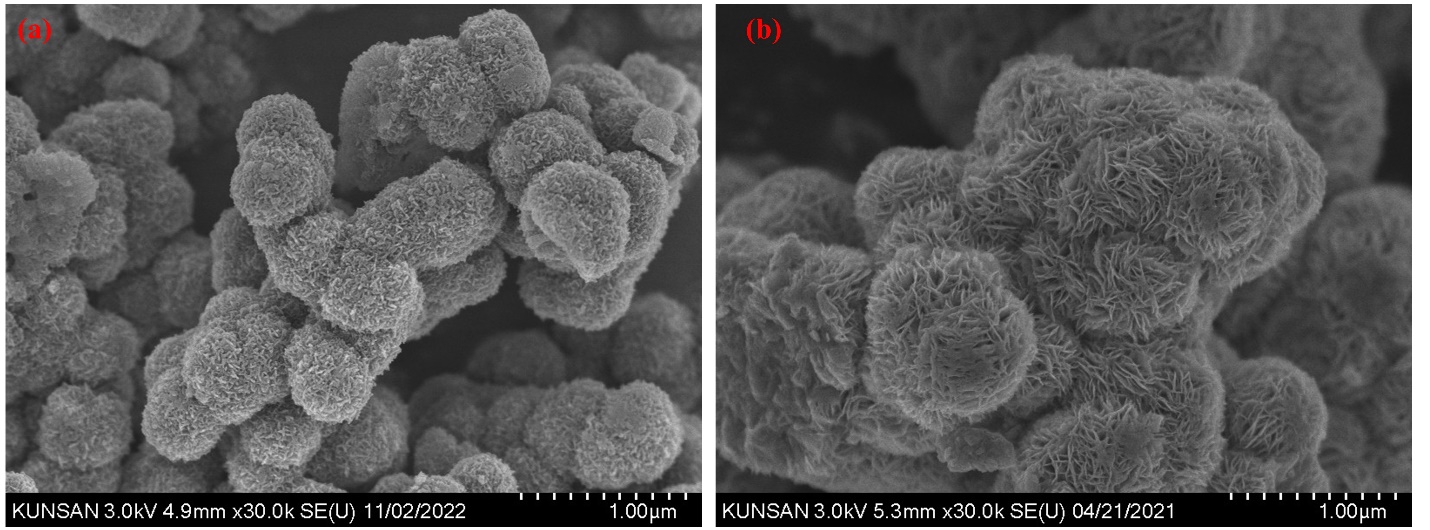


**Fig. S4.** FESEM images (a) Cu@Pani and (b) Cu@Pani/MoS_2_.
